# The Macular Choriocapillaris Flow in Glaucoma and Within-Day Fluctuations: An Optical Coherence Tomography Angiography Study

**DOI:** 10.1167/iovs.62.1.22

**Published:** 2021-01-21

**Authors:** Paolo Milani, Lara Enrica Urbini, Ennio Bulone, Ugo Nava, Deborah Visintin, Giorgia Cremonesi, Lorenza Scotti, Fulvio Bergamini

**Affiliations:** 1 Ophthalmology Unit, IRCCS Istituto Auxologico Italiano, Milan, Italy; 2 Translational Medicine Department, Eastern Piedmont University, Novara, Italy

**Keywords:** glaucoma, optical coherence tomography (OCT) angiography, choriocapillaris

## Abstract

**Purpose:**

To assess quantitatively the choriocapillaris (CC) perfusion area in the macular area of healthy eyes, eyes with primary open-angle glaucoma, and eyes with ocular hypertension using optical coherence tomography angiography (OCTA).

**Methods:**

A consecutive series of healthy individuals and patients with glaucoma and ocular hypertension were recruited prospectively in this single-center, cross-sectional study based in Milan, Italy. OCTA was performed in the morning and evening, along with a complete ophthalmologic examination. Macular superficial capillary plexus vessel density (SCP-VD) and the thicknesses of the retina and ganglion cell complex (GCC), as well as their fluctuations, were investigated.

**Results:**

Thirty-nine eyes from 24 individuals with glaucoma (mean age = 58.79 ± 6 years), 43 eyes from 27 individuals with ocular hypertension (59.19 ± 6 years), and 54 eyes from 35 controls (58.27 ± 6 years) were enrolled. The mean CC perfusion area values were not significantly different among the three groups in the morning or evening (*P* ≥ 0.47). In contrast, SCP-VD, retinal thickness, and GCC thickness were statistically different among the groups (*P* ≤ 0.016), except for the foveal SCP-VD (*P* ≥ 0.19) and the evening foveal thickness (*P* = 0.57). Diurnal changes in the CC perfusion area, SCP-VD, retinal thickness, and GCC thickness were not statistically significant (*P* ≥ 0.16). Systemic hypertension, sex, age, axial length, and diurnal changes in intraocular pressure were not significantly associated with morning or evening measurements, or with diurnal fluctuations (*P* ≥ 0.07).

**Conclusions:**

The macular CC flow perfusion area appears unaffected in eyes with primary open-angle glaucoma. No significant diurnal changes were observed in any of the parameters investigated.

Optical coherence tomography angiography (OCTA) enables the investigation of vascular pathogenesis in several retinal diseases. It is now commonly accepted that microvascular perfusion of tissues is strongly related to vessel density (VD), which can be mapped using commercially available OCTA systems.^1^ Hence, the macular and papillary areas are usually studied by visualizing its well-delineated vasculature, which is clearly distinguishable from the surrounding tissue. In the macula, the superficial vascular plexus is located at the level of the ganglion cell layer. The choriocapillaris (CC) is located in the innermost layer of the choroid and is organized as a highly anastomosed network of capillaries, comprised of numerous small end arteries.^2^ Thus, differently from the overlying vascular plexuses, the CC is typically visualized, on OCTA, as a granular image constituted by small dark and bright adjacent lobular areas that correspond to the absence or presence of blood flow, respectively.^3^ In the peripapillary area, OCTA can be used to visualize the superficial, radial capillary plexus of the optic nerve head, which extends from the internal limiting membrane to the outer limit of the retinal nerve fiber layer (RNFL); it is composed of the vasculature of the RNFL and the ganglion cell complex (GCC).

Substantial evidence of reduced perfusion in the peripapillary superficial VD^4^^–^^7^ and in the macular VD^8^^–^^10^ has been reported in individuals with primary open-angle glaucoma (POAG). In addition, the peripapillary CC is typically affected by high intraocular pressure (IOP), and complete atrophy, also known as deep-layer microvascular dropout, frequently occurs.^11^^–^^13^ Despite these important insights in the peripapillary area, alterations in macular CC have been much less studied in glaucoma and with conflicting results.^10^^,^^14^^,^^15^ Investigation of the macular choroid is gaining interest in the pathogenesis of glaucoma because it has a dynamic vascular structure and its thickness can be reduced in glaucomatous eyes.^16^^,^^17^ In the macula, choroidal and CC thickness have been reported to exhibit statistically significant diurnal fluctuations in healthy eyes, as determined using OCTA^18^^,^^19^; however, to the best of our knowledge, no study has conducted similar investigations in eyes with glaucoma.

Therefore, in this study, we aimed to use OCTA for the quantitative assessment of the CC vascular perfusion in the macular area of eyes with POAG, eyes with ocular hypertension, and healthy eyes. Macular superficial capillary plexus vessel density (SCP-VD), as well as retinal and GCC thickness were investigated, along with their morning to evening fluctuations. Finally, linear relationships among parameters, and relationships of the fluctuations with IOP changes, axial length, systemic hypertension, imaging quality, age, and sex were evaluated.

## Methods

### Study Population

A consecutive series of patients with glaucoma, patients with ocular hypertension, but without visual field deterioration, and healthy controls were recruited prospectively between March 2018 and February 2019. This institutional, single-center study was conducted in the Department of Ophthalmology, IRCCS Istituto Auxologico Italiano, Milan, Italy. It was approved by the Institutional Review Board of the Institute (protocol number: 2018 01 30 07) and adhered to the tenets of the 1964 Helsinki Declaration and its later amendments. Informed consent was obtained from each participant included in the study.

The inclusion criteria for all eligible participants were as follows: age between 40 and 70 years, presence of an open iridocorneal angle on gonioscopy, and a refractive error between −2.5 and +2.5 diopters. The exclusion criteria for all individuals were as follows: the presence of any other macular or optic nerve disease; previous eye surgery, other than uneventful cataract surgery but not in the 6 months prior to enrollment; an inability to fixate; an incapacity to provide informed consent; considerable media opacity preventing adequate image quality; and diabetes.

The inclusion criteria for eyes with glaucoma were: IOP ≥ 21 mm Hg in 3 consecutive visits prior to topical treatment; repeatable glaucomatous visual field damage defined as a Glaucoma Hemifield Test (GHT) outside normal limits and a pattern standard deviation (PSD) outside 95% normal limits; glaucomatous visual field abnormalities defined by a cluster of +3 adjacent points in the pattern deviation plot with a probability of < 5%, including at least one point having a probability of < 1% in at least 2 repeatable and consecutive standard automated perimetry tests.

The inclusion criteria for eyes with ocular hypertension were: IOP ≥ 21 mm Hg in 3 consecutive visits prior to topical treatment; normal-appearing optic disc, and intact neuroretinal rim on clinical examination defined as a vertical cup-to-disc ratio (CDR) < 0.5 and a vertical CDR asymmetry ≤ 0.2, with the minor vertical CDR ≤ 0.4; normal visual fields, defined as a PSD within 95% confidence limits, and a GHT result within normal limits average and quadrant RNFL thickness within 95% and 99% confidence limits, respectively.

The inclusion criteria for eyes in the control group included individuals without ocular hypertension or glaucoma with the following characteristics: IOP < 21 mm Hg without therapy; no history of elevated IOP: normal-appearing optic disc and intact neuroretinal rim on clinical examination defined as a vertical CDR < 0.5 and a vertical CDR asymmetry ≤ 0.2, with the minor vertical CDR ≤ 0.4; normal visual fields, defined as a PSD within 95% confidence limits and a GHT result within normal limits; average and quadrant RNFL thickness within 95% and 99% confidence limits, respectively.

Eyes with normal tension glaucoma were excluded, whereas presence of systemic hypertension was noted.

### Study Design and Imaging Acquisition

A complete ophthalmologic examination was performed on all the participants, including slit-lamp biomicroscopy, ultrasound pachymetry, gonioscopy, and dilated-fundus examination. Goldmann applanation tonometry was performed at 8 a.m. and 7 p.m. and axial length was measured at 8 a.m. for every eye using the IOL Master 500 (Carl Zeiss, Jena, Germany). Each participant also underwent standard automated perimetry (24-2 Swedish Interactive Threshold Algorithm; Humphrey Field Analyzer II; Carl Zeiss Meditec, Inc., Dublin, CA, USA) and OCTA.

The OCTA system used for this study was the XR Avanti device with the AngioVue imaging system and AngioAnalytics software version 2017.1.0.150 (Optovue Inc., Fremont, CA, USA), which enables the visualization of the retinal and papillary circulation through a split-spectrum amplitude-decorrelation angiography (SSADA) algorithm. This instrument has a rate of 70,000 A-scans per second, using a light source centered on 840 nm.

Two of the authors (D.V. and G.C.), with a wide experience in OCTA acquisition, used the “AngioRetina” 3 × 3-mm acquisition module at 8 a.m. and 7 p.m. This macular scanning protocol consists of 2 orthogonal OCTA volumes, centered in the fovea, containing 304 × 304 scans to perform motion correction and minimize motion artifacts arising from microsaccades and fixational changes. In this study, the following two retinal slabs, generated automatically by the software, were considered: the SCP (from the inner limiting membrane to the inner plexiform layer) and the CC (from the superior boundary set at 9 microns anteriorly to Bruch's membrane and the inferior boundary set at 31 microns posteriorly to Bruch's membrane). For the SCP, the complete image generated by the software was considered, as well as the foveal and parafoveal subsections. The foveal zone is a small circle of 1-mm diameter automatically centered on the foveola by the software integrated in the device. The parafoveal area is the area between the 1 and 3 mm concentric rings centered on the foveola. Vascularity data are automatically converted to a VD map, in which the VD is calculated as the percentage area occupied by blood vessels as detected by the SSADA technology of the device. The percentage area is calculated automatically for the total macular, foveal, and parafoveal SCP. The corresponding macular retinal thickness is also generated automatically. Centering on the foveola is essential to ensure the accuracy of VD and retinal thickness evaluation, and it was verified carefully during both the acquisition and reading processes.

Blood flow in the CC was calculated (in mm^2^) using the automatic “flow” analysis function in a selected area of 3,144 mm^2^ centered on the fovea. The built-in analysis software uses a binary mask to calculate the blood perfusion (white pixels) versus nonperfusion (black pixels) area (in mm^2^) within the aforementioned circular region. Because the software does not use volumetric parameters over units of time, but area measurements in square millimeters, we think it is more appropriate in this study to cite the CC flow in terms of CC vascular perfusion area.

For the calculation of GCC thickness (i.e. the combined thickness of the RNFL, ganglion cell layer, and inner plexiform layer) the appropriate scanning protocol was used. The center of the GCC scan is automatically shifted temporally by 0.75 mm to improve sampling of the temporal periphery. The resulting map shows the averaged thickness (in microns) of the total GCC, the hemi-superior section, and the hemi-inferior section.

Two of the study authors (P.M. and L.U.) evaluated the automatically generated slabs and excluded altered segmentations or persistent artifacts. They checked each B-scan in the entire macular area to verify the correct localization of the segmentation boundaries, although the software had an automatic “projection artifact removal” functionality. To avoid bias due to the customized adjustment of slabs, eyes with incorrect segmentation were excluded immediately from the analysis. Only high-quality images, defined as scans with a quality ≥ 8/10, were considered.

### Statistical Analysis

Descriptive statistics were calculated to summarize the main demographic and clinical characteristics of the study participants. Categorical variables are reported as frequencies and percentages, and continuous variables as means and standard deviations (SDs).

The χ^2^ test was used to assess the association between systemic hypertension and sex at a patient level and group level (control, ocular hypertension, or glaucoma). Differences in age among the groups were assessed using 1-way ANOVA. Differences in axial length and ocular pressure (both measured in the morning and in the evening, as well as the change between the two measurement time points), as well as in the mean deviation and PSD, were assessed (per eye) using repeated-measures ANOVA to account for within-subject correlation of parameters. To evaluate the differences in the average CC perfusion area in the macular area (measured in the morning and the evening, as well as the morning to evening change) among healthy, hypertensive, and glaucomatous eyes, three linear mixed models were fitted. The response variable of each model was the CC perfusion area, measured in either the morning or the evening, or the morning to evening change; the independent variables were the group, sex, systemic hypertension, age, axial length, and morning to evening changes in ocular pressure. The same models were fitted using either the macular SCP-VD, retinal thickness, or GCC thickness as the response variables. Given the large number of tests performed, when suitable (i.e. when at least one null hypothesis was rejected), false discovery rate (FDR) adjustment was applied to the ANOVA tests. To evaluate the linear correlation between the CC perfusion area (measured in the morning and the evening, as well as the morning to evening change) and the corresponding measurements of the macular SCP-VD, retinal thickness, and GCC thickness, Spearman's correlation coefficients were calculated for all participants and stratified by healthy, hypertensive, and glaucomatous eyes. The *P* values of the tests for absence of correlation were also reported. The values of the parameters measured in the left and right eyes of the same patients were averaged before calculation of the correlation coefficients. R values lower than 0.4 were suggestive of a weak correlation; between 0.4 and 0.6 indicated a moderate correlation; and higher than 0.6, a strong correlation.

The power calculation of the statistical analysis was assessed as follows: assuming a type I error of 0.05, a sample size of 136 eyes, an average daily change in CC perfusion area of −0.027, 0.004 and 0.014 in healthy (*n* = 54), hypertensive (*n* = 43), and glaucomatous (*n* = 39) eyes, respectively, and a common SD of 0.068, it was possible to reject the null hypothesis of the F test for the equality of means with a power of 0.8.

All analyses were performed using SAS (SAS Institute, Cary, NC, USA), all statistical tests were 2-tailed, and a *P* value < 0.05 was considered statistically significant.

## Results

Thirty-nine eyes from 24 individuals with glaucoma (mean age = 58.79 ± 6 years), 43 eyes from 27 individuals with ocular hypertension (mean age = 59.19 ± 6 years), and 54 eyes from 35 controls (mean age = 58.27 ± 6 years) were included in this study. Twenty-six eyes from 13 individuals were excluded because of unsatisfactory automatic segmentation and/or imaging quality.

No differences were discovered among groups in terms of age, sex, axial length, or the presence of systemic hypertension (Table 1; *P* ≥ 0.23). Differences were observed for IOP measured in the morning (*P* = 0.0002) and evening (*P* < 0.0001), but not for the change in IOP throughout the day (*P* = 0.19).

**Table 1.** Demographic and Clinical Characteristics of the Patients Included in the Study

	Total	Normal Control	Hypertension	Glaucoma	
	*N* = 86	*N* = 35	*N* = 27	*N* = 24	
Variables	136 Eyes	54 Eyes	43 Eyes	39 Eyes	*P* Value
Sex, *N* (%)					
Females	48 (56.98)	18 (51.53)	15 (55.56)	16 (66.67)	0.5013^a^
Males	37 (43.01)	17 (48.57)	12 (44.44)	8 (33.33)	
Systemic hypertension, *N* (%)					
No	67 (77.91)	25 (71.43)	24 (88.89)	18 (75.00)	0.2389^a^
Yes	19 (22.09)	10 (28.57)	3 (11.11)	6 (25.00)	
Age, y (mean, SD)	58.27 (6.75)	57.20 (6.68)	59.19 (6.69)	58.79 (6.70)	0.4735^b^
Axial length, mm (mean, SD)	23.72 (1.08)	23.82 (1.08)	23.39 (0.84)	23.95 (1.24)	0.8420^c^
Ocular pressure, morning, mm Hg (mean, SD)	16.79 (3.42	15.63 (2.72)	19.00 (3.44)	15.97 (3.16)	**0.0002** **^c^**
Ocular pressure, evening, mm Hg (mean, SD)	15.38 (3.24)	14.75 (2.24)	17.50 (3.50)	13.95 (3.00)	**<0.0001** **^c^**
Diurnal change in ocular pressure, mm Hg (mean, SD)	1.37 (2.31)	0.87 (2.07)	1.38 (2.54)	2.03 (2.27)	0.1963^c^
Mean deviation, dB (mean, SD)	−1.57 (3.76)	−0.75 (1.32)	−0.53 (1.25)	−3.92 (6.22)	**0.0038** **^c^**
Pattern standard deviation, dB (mean, SD)	2.10 (1.77)	1.66 (0.62)	1.61 (0.45)	3.27 (2.92)	**0.0002** **^c^**

a *P* values from χ^2^ test.

b *P* values obtained from ANOVA test.

c *P* values obtained from repeated measure ANOVA.

Statistically significant values are in bold.

Table 2 displays the means and corresponding SDs of the CC perfusion areas and the other ocular parameters measured in the morning and evening, as well as the FDR-adjusted *P* values of the ANOVA test for intergroup differences derived by the multivariate model. The mean CC perfusion area values were not significantly different among the three groups in the morning or evening (*P* ≥ 0.47). In contrast, the mean measurements of SCP-VD, GCC thickness, and retinal thickness were statistically different among the groups (*P* ≤ 0.016), except for the foveal SCP-VD (*P* = 0.19) and the evening foveal thickness (*P* = 0.57). Table 3 indicates the corresponding morning to evening changes in the three groups of patients and the *P* value of the ANOVA test obtained from the multivariate model. Neither the CC nor the other parameters investigated exhibited any statistically significant morning to evening variation (*P* ≥ 0.16). Even when comparing glaucomatous and nonglaucomatous eyes (healthy eyes + eyes with ocular hypertension), there were no statistically significant differences in CC perfusion area measured in the morning and evening, or in morning to evening variations (data not shown, *P* ≥ 0.36).

**Table 2.** Means and Corresponding SDs of the Ocular Parameters Measured in the Morning and in the Evening in the Three Groups of Eyes

	Morning				Evening			
	Normal Control	Hypertension	Glaucoma	FDR Adjusted	Normal Control	Hypertension	Glaucoma	FDR Adjusted
	Mean (SD)	Mean (SD)	Mean (SD)	*P* Value	Mean (SD)	Mean (SD)	Mean (SD)	*P* Value
Macular CC perfusion area flow, mm^2^	2.110 (0.110)	2.117 (0.111)	2.125 (0.110)	0.4745	2.137 (0.099)	2.103 (0.103)	2.120 (0.103)	0.5471
SCP-VD (%)								
Whole-SCP-VD	51.337 (2.602)	50.781 (2.783)	45.726 (4.669)	**<0.0001**	51.504 (2.073)	50.812 (2.907)	46.069 (4.889)	**<0.0001**
Fovea-SCP-VD	21.674 (7.753)	23.063 (6.792)	18.210 (9.034)	0.1902	21.609 (7.887)	22.721 (6.228)	18.774 (8.681)	0.5855
Para-fovea-SCP-VD	54.052 (3.801)	53.472 (2.786)	49.687 (4.582)	**0.0005**	54.037 (2.498)	53.300 (3.609)	50.154 (4.692)	**0.0022**
Macular thickness, µm								
Whole thickness	282.074 (9.491)	282.86 (12.813)	266.923 (14.516)	**0.0006**	281.685 (9.453)	282.767 (13.25)	266.282 (14.907)	**0.0002**
Fovea thickness	257.611 (20.137)	264.628 (30.236)	247.897 (20.758)	**0.0165**	258.333 (19.527)	262.744 (23.044)	248.128 (21.942)	0.5769
Para fovea thickness	322.296 (11.424)	324.512 (14.023)	308.564 (18.519)	**0.0108**	321.63 (11.42)	324.14 (14.669)	307.923 (18.987)	**0.0059**
GCC thickness, µm								
Whole GCC	95.333 (4.911)	94.000 (5.831)	81.667 (12.244)	**<0.0001**	95.259 (4.938)	93.791 (6.205)	81.821 (12.314)	**<0.0001**
Hemi inferior GCC	94.796 (5.360)	93.186 (6.013)	81.59 (13.226)	**<0.0001**	94.704 (5.417)	93.326 (6.232)	81.564 (13.143)	**<0.0001**
Hemi superior GCC	96.056 (4.854)	94.744 (6.184)	82.128 (12.842)	**<0.0001**	95.704 (4.924)	94.395 (6.329)	81.641 (13.496)	**<0.0001**

The mean measurements of the CC perfusion area flow were not statistically different among the three groups either in the morning or in the evening. The mean measurements of all the other parameters investigated were statistically different among the three groups either in the morning or in the evening, except for the F-SCP-VD and the fovea-thick in the evening. The *P* values were obtained by linear mixed model, including as response variables the SCP-VD, the retinal and GCC thickness parameters, and as covariates the group, sex, systemic hypertension, age, axial length, and morning to evening changes in intraocular pressure. FDR-adjusted *P* values of the analysis of variance test for every parameter are shown.

CC, choriocapillaris; SCP-VD, superficial capillary plexus vessel density; GCC, ganglion cell complex; FDR, false discovery rate.

Statistically significant *P* values are in bold.

**Table 3.** Means and Corresponding SDs Morning to Evening Diurnal Changes of the Ocular Parameters in the Three Groups of Eyes

	Morning to Evening Change			
	Normal Control	Hypertension	Glaucoma	
	Mean (SD)	Mean (SD)	Mean (SD)	*P* Value
Macular CC perfusion area flow (mm^2^)	−0.027 (0.072)	0.014 (0.068)	0.004 (0.068)	0.1624
SCP-VD (%)				
Whole-SCP-VD	−0.167 (2.405)	−0.030 (2.827)	−0.344 (3.540)	0.3931
Fovea-SCP-VD	0.065 (2.752)	0.342 (2.489)	−0.564 (3.396)	0.5020
Para fovea-SCP-VD	0.015 (4.053)	0.172 (3.380)	−0.467 (4.809)	0.2327
Macular thickness, mm^2^				
Whole thickness	0.389 (1.366)	0.093 (1.729)	0.641 (1.513)	0.6317
Fovea thickness	−0.722 (2.302)	1.884 (15.489)	−0.231 (3.717)	0.6308
Para fovea thickness	0.667 (1.467)	0.372 (1.76)	0.641 (2.345)	0.7804
GCC thickness, mm^2^				
Whole GCC	0.074 (1.831)	0.209 (1.536)	−0.154 (1.479)	0.3884
Hemi inferior GCC	0.352 (2.039)	0.349 (1.557)	0.487 (2.553)	0.9126
Hemi superior GCC	0.093 (1.886)	−0.140 (1.627)	0.026 (1.246)	0.5351

The mean morning to evening changes were not statistically significant among the three groups of eyes. The *P* values were obtained by linear mixed model including as response variables the superficial, the retinal, and GCC thickness parameters with the corresponding morning to evening changes, and as covariates the group, sex, systemic hypertension, age, axial length, and morning to evening changes in IOP. *P* values of the analysis of variance test for every parameter are shown.

CC, choriocapillaris; SCP-VD, superficial capillary plexus vessel density; GCC, ganglion cell complex.

Table 4 lists the regression coefficients and corresponding standard error and *P* values of the potential confounders considered in the model used to assess the relationship between the groups and the morning to evening changes in the ocular parameters. FDR adjustment was applied to the *P* values of sex, age, and axial length. Sex, systemic hypertension, age, axial length, and morning to evening changes in IOP were not significantly associated with measurements obtained in the morning or evening (data not shown), or with morning to evening fluctuations (*P* ≥ 0.07).

**Table 4.** Regression Coefficients (β) and Corresponding SE and Value of the Potential Confounders Considered in the Model Used to Assess the Relationship Among Groups and the Morning to Evening Changes for Every Ocular Parameter

	Sex(Females Versus Males)		Age		Systemic Hypertension		Axial Length		Diurnal Changes in IOP	
	β (SE)	*P* Value^*^	β (SE)	*P* Value^*^	β (SE)	*P* Value	β (SE)	*P* Value^*^	β (SE)	*P* Value
Macular CC perfusion area flow	0.001 (0.012)	0.9148	0.001 (0.001)	0.5586	−0.016 (0.013)	0.2433	−0.001 (0.006)	0.9775	−0.001 (0.002)	0.7321
SCP-VD										
Whole-SCP-VD	1.233 (0.525)	0.0713	0.084 (0.041)	0.2148	0.235 (0.602)	0.6971	0.398 (0.269)	0.2799	0.134 (0.111)	0.2286
Fovea-SCP-VD	0.349 (0.541)	0.7444	−0.029 (0.042)	0.6144	0.016 (0.622)	0.9799	−0.216 (0.275)	0.5436	−0.070 (0.116)	0.5479
Para fovea-SCP-VD	1.766 (0.721)	0.0713	0.092 (0.056)	0.3424	−0.138 (0.821)	0.8670	0.524 (0.374)	0.2799	0.156 (0.149)	0.2978
Macular thickness										
Whole thickness	−0.214 (0.324)	0.7444	0.057 (0.025)	0.2148	0.141 (0.378)	0.7109	0.146 (0.156)	0.5024	−0.004 (0.058)	0.9397
Fovea thickness	−0.242 (0.967)	0.8923	−0.013 (0.075)	0.8643	−0.542 (1.071)	0.6138	0.015 (0.530)	0.9775	0.055 (0.204)	0.7896
Para fovea thickness	−0.197 (0.388)	0.7662	0.034 (0.030)	0.5303	−0.139 (0.453)	0.7594	0.26 (0.187)	0.2799	−0.072 (0.075)	0.3348
GCC thickness										
Whole GCC	0.764 (0.299)	0.0713	0.020 (0.023)	0.5586	0.007 (0.347)	0.9842	0.291 (0.148)	0.2641	−0.110 (0.064)	0.0886
Hemi inferior GCC	0.286 (0.348)	0.7444	0.010 (0.027)	0.8063	−0.351 (0.402)	0.3862	0.41 (0.174)	0.2111	−0.076 (0.076)	0.3234
Hemi superior GCC	0.588 (0.281)	0.0984	0.025 (0.022)	0.5303	0.220 (0.326)	0.5014	0.217 (0.137)	0.2799	−0.033 (0.060)	0.5850

The sign minus means an inverse relationship of the corresponding couple of values.

* FDR adjusted *P* values.

Gender, age, systemic hypertension, axial length, and daily changes in intraocular pressure were not significantly associated with the morning to evening changes of the listed parameters.

CC, choriocapillaris; SCP-VD, superficial capillary plexus vessel density; GCC, ganglion cell complex.

For all eyes, the Spearman correlation coefficients (*r*) and the corresponding *P* values of the tests for the absence of correlation among the morning to evening fluctuations of CC perfusion area, SCP-VD, retinal, and GCC thickness are presented in Table 5. Most of the tests showed no statistical significance. The strongest, though weak, correlation was found between the CC and the parafovea SCP-VD in healthy eyes (*r* = −0.356, *P* = 0.000).

**Table 5.** Spearman Correlation Coefficients (*r*) and Corresponding FDR Adjusted *P* Value for the Test to Evaluate Absence of Correlation Between Morning to Evening Changes in Macular CC Perfusion Area Flow and in Macular SCP, Macular Retinal Thickness and GCC Parameters for the Three Groups of Eyes and for the Totality of Eyes

Macular CC Perfusion Area Flowmorning to Evening Changes	Whole SCP-VD	Foveal SCP-VD	Para Foveal SCP-VD	Whole Retinal Thickness	Fovea Thickness	Para Foveal Thickness	Whole GCC Thickness	Hemi Inferior GCC	Hemi Superior GCC
All subjects	−**0.183 (0.008)**	−0.168 (0.015)	−**0.285 (0.004)**	−0.087 (0.681)	0.065 (0.410)	−0.121 (0.621)	−0.012 (0.886)	−0.014 (0.861)	−0.113 (0.741)
Normal control	−**0.236 (0.002)**	−0.080 (0.085)	−**0.356 (0.000)**	−0.088 (0.691)	**0.294 (0.004**)	−0.238 (0.270)	0.044 (0.796)	0.122 (0.510)	−0.129 (0.652)
Hypertension	−0.127 (0.070)	−0.153 (0.087)	−**0.281 (0.016)**	0.141 (0.869)	−0.276 (0.283)	0.006 (0.761)	0.000 (0.586)	−0.177 (0.291)	−0.117 (0.521)
Glaucoma	−0.077 (0.321)	−0.096 (0.378)	−0.160 (0.209)	−0.178 (0.491)	0.013 (0.691)	−0.060 (0.756)	−0.183 (0.485)	−0.033 (0.481)	−0.048 (0.516)

R values lower than 0.4 were suggestive of a weak correlation. The sign minus means an inverse correlation of the corresponding couple of values.

CC, choriocapillaris; SCP-VD, superficial capillary plexus vessel density; GCC, ganglion cell complex.

Statistically significant values are in bold.

## Discussion

**Figure 1.**
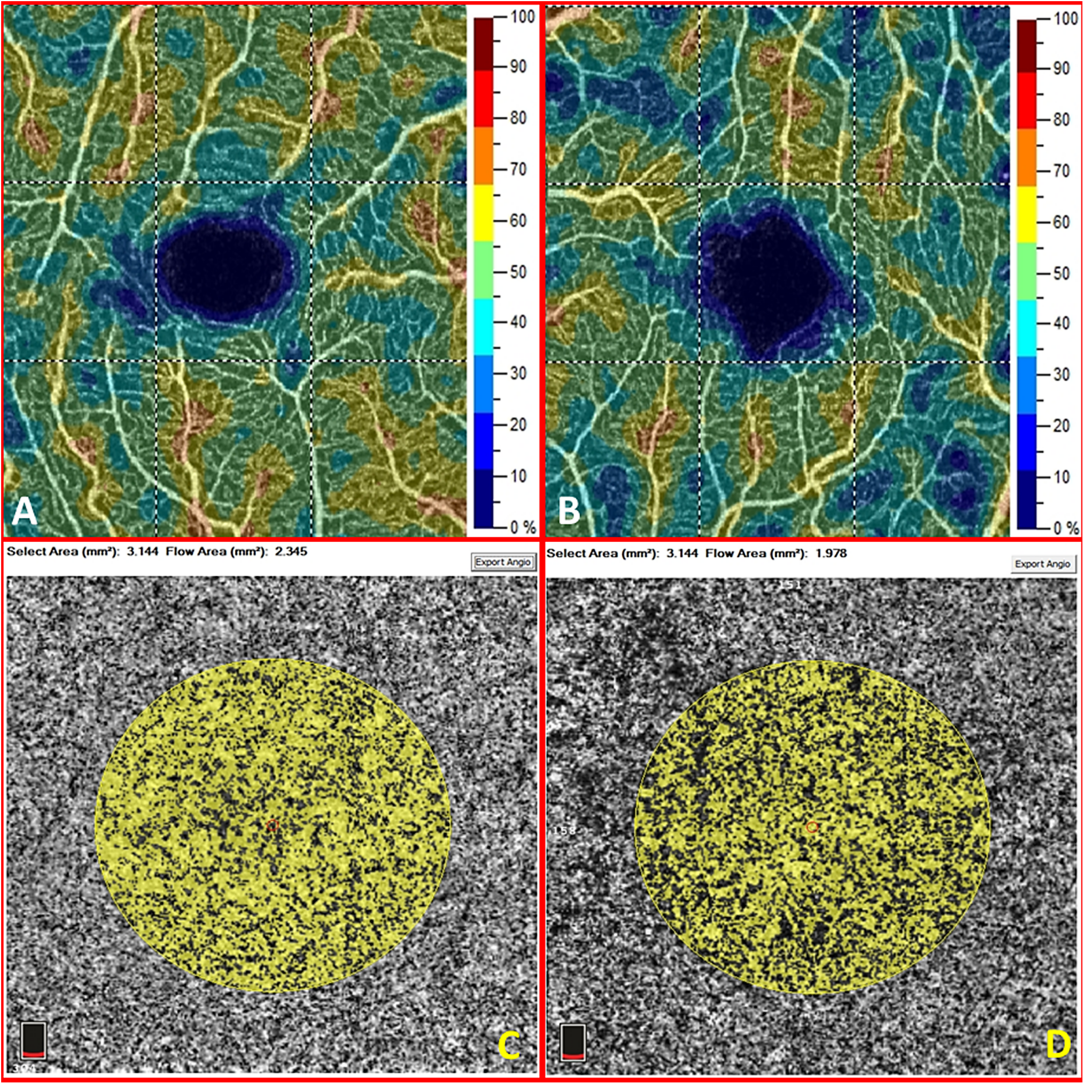
The macular superficial capillary plexus vessel density map (**A**) and the macular choriocapillaris vascular perfusion area map (**C**) of the right eye in a healthy 66-year-old woman. The macular superficial capillary plexus vessel density map (**B**) and the macular choriocapillaris vascular perfusion area map (**D**) of the left eye in a 55-year-old woman with glaucoma. The corresponding whole image vessel density and retinal thickness are significantly reduced in eyes with glaucoma (49.3 vs. 44.1% and 326 vs. 287 microns in this case). In the selected area of 3.144 mm^2^ centered in the fovea, the choriocapillaris perfusion area is reduced in this case (2.345 vs. 1.978 mm^2^). This difference, however, is not statistically significant when considering all glaucomatous eyes of the study versus the others.

**Figure 2.**
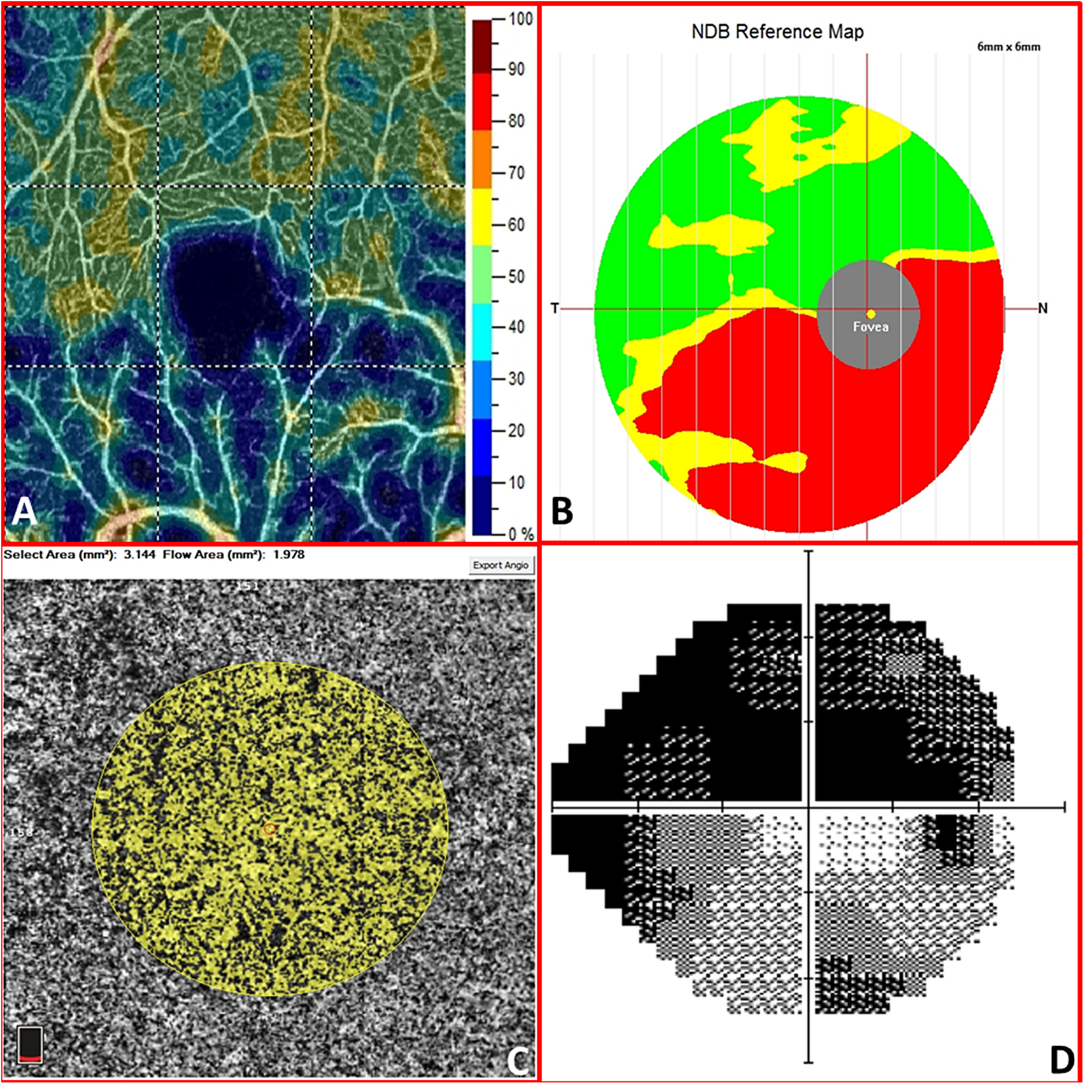
The macular superficial capillary plexus vessel density map (**A**) and the macular choriocapillaris vascular perfusion area map (**C**) of the right eye in a 52-year-old man with advanced glaucoma. The hemi-inferior ganglion cell complex thickness (**B**) is reduced in respect with the normative data base (NDB). This reduction corresponds to a lower vessel density in the hemi-inferior parafovea sector and to the superior scotoma in the computed visual field (**D**). Mean deviation is −22.54 dB, and pattern standard deviation is 9.12 dB.

Our study provides both static and dynamic information. From a static point of view, the macular CC flow (in terms of vascular perfusion area) does not seem to be affected by vascular alterations related to glaucomatous pathology. Thus, unlike the peripapillary and macular VD, no differences could be reported between healthy and glaucomatous eyes regarding the CC flow, according to our study, neither in the morning nor in the evening measurements (Fig. 1). This observation is remarkable because there were statistically significant differences in the density of the overlying retinal vasculature among the three groups of eyes. In fact, the macular retinal thickness, the GCC, and the SVP-VD were substantially reduced in the glaucomatous eyes (*P* < 0.015, except for the foveal SCP-VD). Hence, no projection artifact should account for interfere with the CC perfusion area quantitative measurement (Fig. 2). In the present study, we were unable to assess whether capillary dropout in the SCP depended on the inner retina or vice versa. Although this remains an unresolved question, it is incontrovertible that retinal and GCC thickness are strictly dependent on the SCP that nourishes the inner layers of the retina. Overall, our findings regarding the macular CC vascular perfusion area are consistent with those of the study by Chao et al.,^14^ in which the same device and software were used. In contrast, Yip et al.^10^ revealed a statistically significant reduction in macular CC flow through the same device but availing of an external software capable of counting the pixels in the image to determine the VD automatically. The current paucity of similar reports limits data comparison. In general, we must consider that information on macular CC in glaucomatous eyes is still poor both because of the location (under the retinal pigment epithelium) and the structure (spongiform tissue) that make the investigation by means of OCTA technology more complex than the vasculature of the overlying retina. Moreover, it is clear that the majority of studies have focused on the papillary area, which is considered to be mainly involved by the glaucomatous disease as several superficial peripapillary microvascular changes have been disclosed,^20^^,^^21^ even before the onset of visual field defects.^22^

The vascularization of both the macular and peripapillary CC is derived from the short posterior ciliary arteries, being completely distinct from that of the superficial retina, which is supplied by the central retinal artery. The CC nourishes the RPE and the outer retina in the macular area, and the prelaminar portion of the optic nerve in the peripapillary area. Peripapillary deep-layer microvascular dropout is well-known in glaucoma^11^^–^^13^; conversely, the macular CC is getting explored more recently by means of OCTA technology, with few and contrasting results.^10^^,^^14^^,^^15^ Using spectral-domain OCT too, studies are seldom comparable in POAG, since some authors found reduced mean choroidal thickness predominantly in the peripapillary than in the macular area^17^^,^^23^^,^^24^ whereas some others did not.^25^^,^^26^ Overall, should our study findings be validated by future reports, it would be of clinical relevance. If the macular CC perfusion remains intact regardless of alterations in the overlying retina and the peripapillary area, the choroidal vascular role in POAG pathogenesis might be reconsidered. In fact, it is difficult to believe that CC vascular impairment in POAG can affect the papillary area without affecting the macular area, as both are nourished by the same arteries. Conversely, in normal-tension glaucoma, the only studies to date by means of OCTA showed notably a significant CC flow reduction in both the macular and peripapillary area,^14^^,^^15^ highlighting the relevant role of slower choroidal hemodynamics in this disease, as formerly evidenced by fluorescein angiography too.^27^ These findings, though limited, might lead to consider the peripapillary choroidal microvasculature dropout in POAG as a consequence rather than a cause of RNFL and GCC rarefaction.

The other information provided by the present study is dynamic. To the best of our knowledge, we are the first to investigate the change in the morning and evening macular CC vascular perfusion area in POAG; we report that there were no statistically significant differences among eyes in any of the examined groups. The diurnal stability of superficial and deep-macular VD, and of the retinal thickness, has already been described.^28^^,^^29^ The retinal vasculature, although reduced in eyes with POAG and ocular hypertension, may be unaffected by diurnal circadian changes, at least to the extent that can be assessed using OCTA. In fact, abnormal autoregulation of ocular blood flow in glaucoma was evidenced using different technologies, such as color/laser Doppler flowmetry,^30^^,^^31^ whereas OCTA can only assess quantitatively the vessel caliber volume, not the volume of blood flow. It must be acknowledged, however, that choroidal thickness varies during the day in healthy eyes, being increased in the morning, probably because of diurnal blood pressure variations.^32^ The morning to evening stability in CC perfusion assessed in the present study does not contradict the generally accepted choroidal metrics. In fact, the CC represents only the inner part of the choroid, whereas the deeper Sattler's and Haller's layers probably account for variations in diurnal thickness, as reported recently by Gabriel and Siegfrid.^19^^,^^33^ These authors were the ones to make the effort to distinguish morphologically from the choroid, reporting that Sattler's layer alone was thicker in the morning. Furthermore, the evidence of choroidal-vessel enlargement (“pachyvessels”) in the pachychoroid spectrum of diseases suggests that the outer part of the choroid, more than the CC, is likely sensible to vascular dynamic changes or altered local circulation.^34^ Consistently, Kinoshita et al. highlighted diurnal variation of luminal rather than stromal tissue metrics in the choroid reporting a significant positive correlation between mean arterial pressure and luminal tissue.^35^ It is also known that the parasympathetic, the sympathetic and the trigeminal fibers innervate the choroid in mammals, but they are localized to the walls of the arteries and veins of the choroid, but not the choriocapillaris.^36^ Therefore, it is possible that the CC remains a stable and independent vascular district within the choroid, whereas the outer portions of the choroid are mostly sensible to systemic pressure changes, diurnal fluctuations, and pathological alterations. To date, only Sarwar and collaborators^18^ were able to disclose a minimal though statistically significant difference between morning and evening CC vascular density, using the same OCTA device, but with built-in software that has now been discontinued by the manufacturer because of low reproducibility.

In our study, sex, systemic hypertension, age, axial length, and morning to evening changes in IOP were not significantly associated with morning and evening measurements or with diurnal fluctuations in CC perfusion, SCP-VD, GCC, or retinal thickness. Unfortunately, we did not measure specific hemodynamic parameters, such as blood pressure. It could have been interesting to confirm the study by Muller and collaborators^37^ findings of a higher arterial pressure being related to an increase in SCP-VD; however, it must be considered that no relationships between CC morning to evening fluctuations and mean arterial pressure were disclosed in another study, at least in healthy eyes.^19^

Despite the amount of data collected, one limitation of our study is the exclusion of the deep capillary plexus from the analysis. However, this vascular complex has been investigated in glaucomatous eyes with controversial findings, and it seems to be unaffected by glaucoma.^38^^,^^39^ Another limitation is the exclusion of the peripapillary portion of the CC and the deeper layers of the choroid from the investigation. Extending the OCTA segmentation to the outer choroid could have disclosed further interesting insights. However, the outer segmentation provided automatically by the XR Avanti device is set 31 microns inferiorly to the RPE. Proceeding deeper into the choroid is an operator-dependent procedure with various possible signal and artifact alterations due to the anatomic nature of the outer choroid. Thus, when designing the study, we preferred to focus on the CC layer, comprising approximately 40 out of 270 microns of the choroid in the submacular area. A substantial limitation concerns the calculation of the CC flow area in mm^2^ rather than as a percentage, according to the spongious rather than vascular nature of the CC. The OCTA device's built-in software has been validated by many studies, although certain authors use external software to convert original images to binary images. Overall, we think that the information derived in mm^2^ is a real vascular parameter, although referring to the CC perfusion area and not the volume. We also acknowledge that, with only two measurements (morning and evening), one cannot exclude CC perfusion alterations throughout the day. It may be argued that the absence of statistically significant alterations in CC perfusion may be related to our study's sample size or to an intrinsic error in the quantification of the CC perfusion by the automatic software. However, a certain interdependence between some parameters in morning to evening increasing or decreasing values was disclosed by the linear relationship's analysis, confirming the validity of our measurements. Most of the Spearman correlation coefficients were not statistically significant, although a weak correlation (*r* < 0.36) was discovered in some instances (see Table 5). Finally, it should be noted that the version of the software used in this study has the projection artifact removal and the motion correction technology to minimize these important features related to the OCTA technology. Although it is possible that the CC could be influenced by the overlying superficial, intermediate, and deeper capillary plexuses, the Avanti system's algorithm should guarantee the removal of the majority of artifacts, even in the deep layers.

In conclusion, the macular CC perfusion area appears to be unaffected in POAG eyes, despite statistically significant differences in SCP-VD, retinal, and GCC thickness. No significant morning to evening changes were disclosed in any of the parameters investigated. Future and larger studies should better elucidate the role of the macular CC in the pathogenesis of glaucoma.
